# Staff’s perspectives on physical activity in acute mental health general adult wards: a follow up survey

**DOI:** 10.1192/j.eurpsy.2023.1154

**Published:** 2023-07-19

**Authors:** N. Lekka, J. Wrazen, S. Nunns

**Affiliations:** Sheffield Health and Social Care Foundation Trust, Sheffield, United Kingdom

## Abstract

**Introduction:**

Physical activity (PA) has multiple health benefits for people with severe mental illness (SMI). People with SMI engage in less exercise and more sedentary behaviour than the general population; this can be further exacerbated by inpatient settings. Staff’s attitudes towards PA may influence patient engagement.

**Objectives:**

In 2019, a study explored staff’s views on PA for acute psychiatric inpatients. This follow-up study by the same team aimed to establish whether the enablers/barriers to promoting PA have changed and to identify targets for intervention.

**Methods:**

In 2022, an online anonymous survey with free text was sent to all multidisciplinary team (MDT) members (n=91) of two acute general adult wards, including nurses, doctors, and allied health professionals (AHPs). A combination of quantitative and qualitative analysis was used to understand participants’ perspectives. Manual thematic analysis was completed to identify discrete themes.

**Results:**

Response rate was significantly lower for the follow-up at 39% as opposed to 63% of the initial study, possibly reflective of post-COVID-19 staffing issues and lack of time for engagement in quality improvement activities. Respondents were nearly unanimous in agreeing that PA was beneficial to physical and mental health. Enablers to PA included higher numbers of staff (24%), more PA resources (22%), more PA-designated staff (19%), more PA-dedicated time (14%), and timetables of available activities (14%). The majority (65%) continued to report that promoting PA was difficult during their shift. Reported barriers included lack of staff (38%), lack of time (27%), and high levels of clinical activity (24%). Noticeably, nurses were much more likely than doctors or AHPs to report short staffing as a barrier to promoting PA (OR=19.8, p < 0.05). Participants described the gym (22%), walking groups (19%), and football (14%) as the most beneficial PA for patients, whilst 14% responded it was “whichever PA patients preferred”. This was mirrored by staff naming “user feedback” as a potential enabler. Reasons for PA being beneficial included “being outside” (24%) and “being inclusive” (11%). Only 45% of MDT members felt they had been provided with PA education/training.

**Conclusions:**

Staff continued to acknowledge the importance of PA for physical and mental health and were aware of multiple enablers and barriers. Post-COVID-19, systemic issues such as staffing levels, lack of time, high levels of clinical activity, and lack of PA education/training remained barriers. Service user preference, enjoying the outdoors and inclusivity were features of activities perceived to be most beneficial. An integrative approach to mental health and wellbeing, providing inclusive activities, educating/training staff, promoting PA in inpatient psychiatric settings, and offering organisational support can contribute to improved PA provision and regular patient engagement.

**Disclosure of Interest:**

None Declared

